# Immune Response Varies with Rate of Dispersal in Invasive Cane Toads (*Rhinella marina*)

**DOI:** 10.1371/journal.pone.0099734

**Published:** 2014-06-17

**Authors:** Gregory P. Brown, Richard Shine

**Affiliations:** School of Biological Sciences, University of Sydney, Sydney, New South Wales, Australia; University of Sao Paulo, Brazil

## Abstract

What level of immunocompetence should an animal maintain while undertaking long-distance dispersal? Immune function (surveillance and response) might be down-regulated during prolonged physical exertion due to energy depletion, and/or to avoid autoimmune reactions arising from damaged tissue. On the other hand, heightened immune vigilance might be favored if the organism encounters novel pathogens as it enters novel environments. We assessed the links between immune defense and long-distance movement in a population of invasive cane toads (*Rhinella marina*) in Australia. Toads were radio-tracked for seven days to measure their activity levels and were then captured and subjected to a suite of immune assays. Toads that moved further showed decreased bacteria-killing ability in their plasma and decreased phagocytic activity in their whole blood, but a heightened skin-swelling response to phytohemagglutinin. Baseline and post-stress corticosterone levels were unrelated to distance moved. Thus, long-distance movement in cane toads is associated with a dampened response in some systems and enhanced response in another. This pattern suggests that sustained activity is accompanied by trade-offs among immune components rather than an overall down or up-regulation. The finding that high mobility is accompanied by modification of the immune system has important implications for animal invasions.

## Introduction

Life histories of many animals incorporate long-distance movements in the form of migration or dispersal [1], [2]. The physical and energetic demands of these movements may shape the morphology, physiology, behavior and energy budgets of the species involved [3], [4]. The response of the immune system to migration or dispersal and to strenuous, sustained physical activity (which accompanies migration/dispersal) recently has attracted research in diverse fields. The observation that strenuous exercise suppresses certain immune functions has enabled investigators to experimentally mimic the immune changes associated with various traumatic and clinical disorders in humans and laboratory animals [5]–[8]. Studies on wildlife diseases and zoonoses have investigated how infection affects the ability of individuals to undertake long-distance movements and thus spread pathogens [3], [9]–[12]. Ecoimmunologists are interested in the energetic and functional trade-offs between immunocompetence and long-distance movements, and the selective forces that shape them [11]–[17]. The links between the immune system and long-distance movements also are an emerging focus of invasion biology [18]–[25].

Long-distance movements could influence immune function via two major pathways. The first arises from the muscular activity involved in the physical act of moving, and the second arises from the array of pathogens that an animal is likely to encounter as a result of moving around. First, muscular activity requires energy and nutrients and thus can alter immune function through resource trade-offs [18], [20], [25]. When sustained strenuous activity is a priority (e.g. during migration or dispersal), resources may be diverted away from other functions, including the immune system [4]. Maintaining a highly responsive immune system is expensive, in part, because of the cost (in energy and nutrients) of producing specialized cells and products [26]–[29]. Thus, when prolonged physical activity is prioritized and alters the energy budget, the immune system may be down-regulated, or the contribution of its various components economized or rationalized [20], [30].

The muscle damage caused by sustained strenuous activity also might down-regulate immune function [30], [31]. Heavy physical exertion can damage cells, thus increasing the presentation of heat shock proteins or increasing the concentrations of other ‘self’ molecules. This situation carries a risk of potentially dangerous autoimmune responses, where the immune system misidentifies and targets ‘self’ antigens. Thus, as an adaptive mechanism to prevent autoimmune reactions, strenuous activity may activate increased production of immunosuppressive neuroendocrine compounds, such as corticosterone [31]. Although the immunosuppression resulting from increased corticosterone levels may be a mechanism to prevent the immune system targeting ‘self’, a consequence may also be a reduced ability to target pathogens in the aftermath of strenuous activity [30], [31].

Further selective forces may emerge when strenuous activity moves the organism into a novel environment and hence, encounters with novel pathogens. The type of selection imposed by novel pathogens depends upon their infectivity and virulence [19], [32], [33]. Because parasites that have not co-evolved with the dispersing organism are unlikely to successfully infect these novel hosts [34], the invader may benefit by dampening its immune responses in ways that curtail unnecessary or inappropriate responses, and the risk of immunopathology [18], [20]. Alternatively, movement into a novel environment may allow the disperser to ‘outrun’ pathogens that occur at the range core and this release from enemies by this means (or by translocation) may in turn decrease demands on, or investment in, the immune system [18], [35].

Most of our information on the relationships between immune responses and long distance movement comes from migratory species, rather than dispersing ones. This distinction is important, because migratory paths and behaviors typically have been shaped over periods long enough for energetic trade-offs and pathogen resistances to have been optimized by selection. Migrating individuals typically do not encounter truly novel environments or pathogens as they move, because they are revisiting areas and pathogens that they (or their ancestors) have encountered before. In addition, migratory events are tightly linked to other factors that may themselves affect immune defense (e.g. reproduction, change in climate or environment, conspecific density, etc.) [36]. Studying immune function among dispersive or range-expanding animals, rather than migratory ones, thus may provide novel insight because the study system includes movement into novel environments and encounters with novel pathogens [14], [16], [23].

Invasive species may provide especially robust opportunities for investigating interactions between strenuous activity, immune function, and encounters with truly novel pathogens or release from prevalent ones [21], [33], [34], [37]. Introduced species can be viewed as experimental manipulations, albeit misguided ones. Animals rapidly expanding into an introduced range, removed from their native-range predators and pathogens, provide a unique opportunity to detect the selective forces that shape trade-offs between long-distance movement and immune function, early in the evolutionary process. As part of an investigation into the ecoimmunology of invasive species, we radio-tracked Australian cane toads and measured their immune function to test the hypotheses that more extensive movements were associated with modified immune function. Further, we assessed whether any changes in immune function were correlated with increased corticosterone levels following strenuous activity (see above).

## Materials and Methods

### Ethics Statement

All procedures were designed to minimize animal suffering, and were approved by the University of Sydney Animal Ethics Committee under protocol L04/1-2010/3/5193.

### Study Species and Area

Cane toads (*Rhinella marina*) are large (to 150 mm snout-urostyle length,  = SUL) bufonid anurans native to Central and South America. They were intentionally introduced to Australia in 1935 to control insect pests in sugar cane crops [38]. Since their introduction, toads have spread at an increasing rate through >1 million hectares of northern and eastern Australia [39]. Radio-tracking studies have shown a remarkable acceleration in the course of the toads’ Australian invasion, with individual toads at the invasion front likely able to achieve movements >50 km per year [40].

The toad invasion reached our study site, 60 km SE of Darwin in Australia’s Northern Territory, in early 2005. Thus, the current study (conducted in 2013) involved free-ranging toads from a population eight years post-invasion. Toads were captured and radio-tracked on a pastoral property (12.64°S, 131.62°E) adjacent to the floodplain of the Adelaide River. The study area is mainly low-lying, consisting of seasonally inundated floodplain, pasture and savannah woodlands around a small group of rocky hills (see [41] for more detail, including maps).

**Figure 1:**
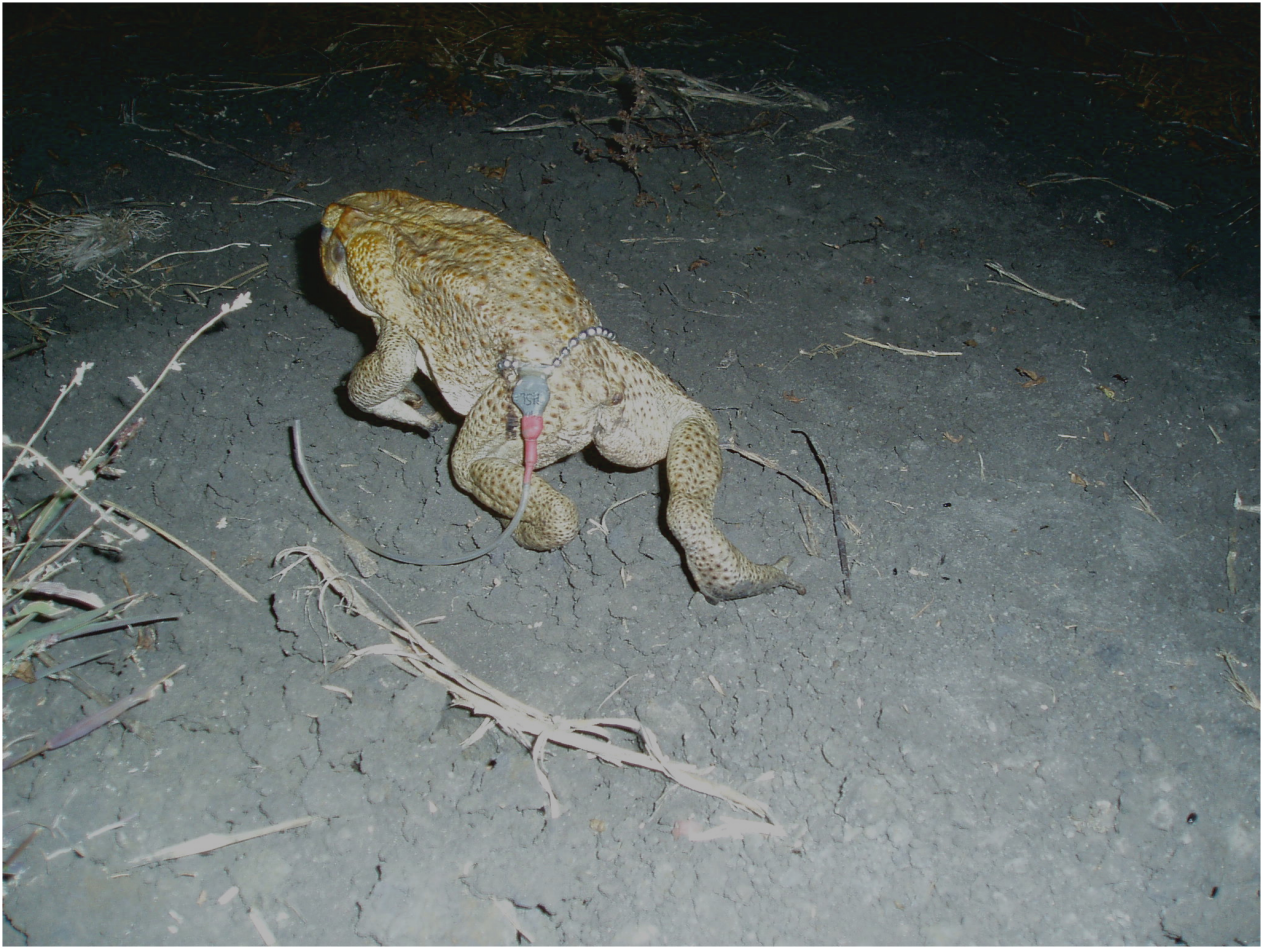
Toad in motion. Female cane toad (*Rhinella marina*) bearing a 3g radio-transmitter on a bead chain belt.

The area experiences a wet-dry tropical climate. Maximum daily air temperatures are high (>32°C) year round but rainfall is largely restricted to the November–April wet season [42]. The present study took place between 3 January and 14 March 2013, the height of the wet season, when environmental conditions are optimal for toad activity and movement [43]. On three nights during this period, we collected toads along a 1.5-km section of dirt road. Toads were returned to the lab and on the following day were weighed, measured for body size (SUL) and sexed (based on skin rugosity and coloration and the presence of nuptial pads and a release call in males). On each of the three capture occasions, 10 toads (5 males and 5 females) were matched for body size as closely as possible, and selected for radio-tracking. The male toads ranged in body size from 89–122 mm SUL and from 92–234 g in mass. Female toads ranged in size from 93–128 mm SUL and from 93–270 g in mass. Each toad was fitted with a 3-g radio-transmitter (model PD2; Holohil Systems, Ottawa, Canada) attached to a bead chain belt (Fig. 1). Toads were released at their capture point during the evening. Over the following week, toads were located each day and their position mapped using a handheld GPS. Toads in tropical Australia are nocturnal and all movements occur at night. Radio-tracking was done during daylight hours and the toads’ locations represent their daytime refugia.

After 7-days of radio-tracking, we recaptured 24 of the 30 toads to perform immune assays on them. Three toads had moved to inaccessible areas and were not captured on schedule, two toads were killed by predators (probably water rats, *Hydromys chrysogaster*) during telemetry, and the transmitter belt fell off one toad. As soon as each toad was located on the seventh day of radio-tracking, it was captured and a 0.3 mL blood sample was collected immediately (within 3 min) via cardiac puncture [44], using a heparinized syringe. Blood samples were placed into sterile 1.5 mL vials and transported back to the laboratory in an ice-water bath to minimize sample degradation. Collecting and blood sampling toads on the final day of telemetry took approximately three hours in total. Immediately upon return to the laboratory, an aliquot of whole blood was removed from the blood sample and used for (1) preparation of a blood smear, (2) hemocytometry, and (3) a phagocytosis chemiluminescence assay. The remaining blood sample was centrifuged at 12000 G for 2 min and the plasma collected. Fresh plasma was used for bacteria-killing assays and the remainder frozen, for later measurement of corticosterone.

In the laboratory, toads were held individually in damp cloth bags, indoors at 25°C. The following morning they were removed from their bags and a second blood sample of 0.1 mL was collected and immediately centrifuged as above, and the plasma collected and frozen for later corticosterone determination. After this second blood sample was collected, we commenced a phytohemagglutinin (PHA) skin-swelling assay on each toad (see below).

### Immunoassays

#### White blood cell differentials and hemocytometry

**Figure 2:**
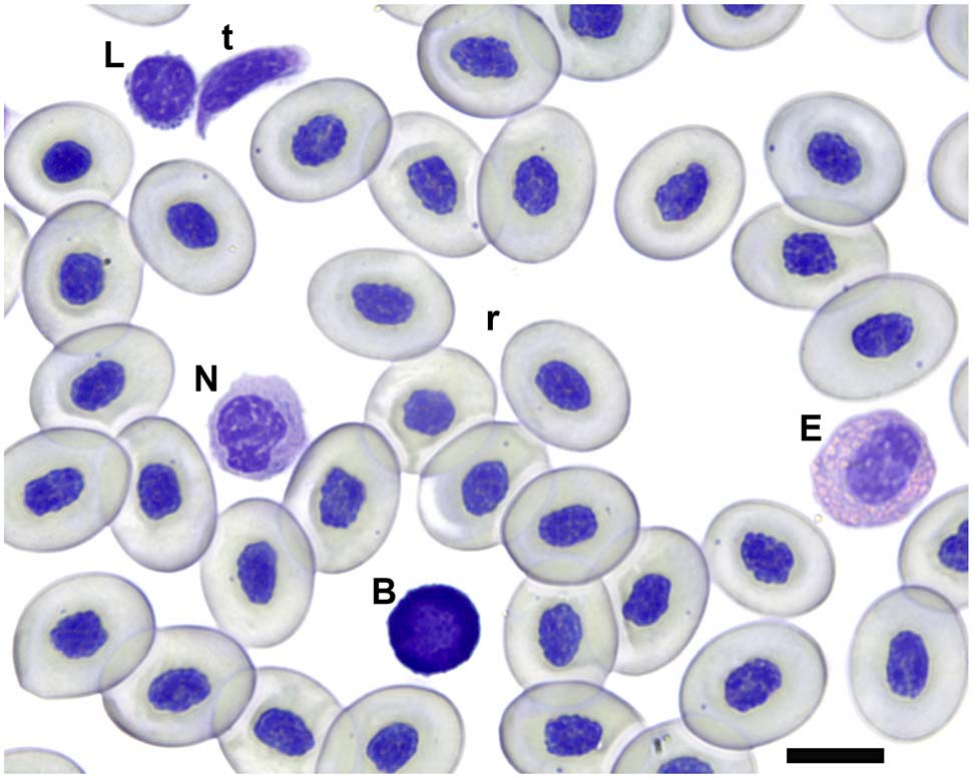
Cane toad blood cells. Giemsa-stained cane toad blood smear with representative selection of blood cell types. Letters to the upper left of cells denote: (B) basophil, (E) eosinophil, (L) lymphocyte, (N) neutrophil, (r) erythrocyte, and (t) thrombocyte. Scale bar in the lower right indicates 10 µm.

We used blood smears to make differential counts of white blood cells, and hemocytometry to determine the concentration of blood cells in circulation. Blood smears were air dried overnight then fixed in methanol and stained using modified Giemsa. They were fitted with a cover glass and examined at 1000X. Slides were scanned and the first 100 white blood cells encountered were identified as basophil, eosinophil, monocyte, neutrophil or lymphocyte (Fig. 2). For hemocytometry, 10 µL of whole blood was added to 990 µL of Natt Herrick solution. The vial was then refrigerated for 60 min, after which time the vial was vortexed and 10 µL of the stained whole blood dilution was used to charge the chamber of a hemocytometer. We then examined the chamber under 400X magnification to count red and white blood cells [44].

#### Bacteria killing assay

Bacterial pathogens are neutralized through the action of soluble proteins (e.g. complement, natural antibody, lysozyme) in plasma [45]. We tested the bactericidal capacity of toad plasma using a modification of Matson et al.’s method [45]. We used two types of bacteria, *Escherichia coli* (ATCC 8739) and *Ochrobactrum anthropi* (ATCC 49687). We chose *O. anthropi* as a microbial challenge because this bacteria is associated with spondylitic pathology in invasive cane toads [21]. Lyophilized pellets of each bacteria were dissolved in sterile phosphate buffered saline (PBS), such that 10 µL contained approximately 900 colony-forming units. Plasma from each toad was diluted 1∶10 in sterile CO_2_-independent media enriched with L-glutamine. 140 µL of the diluted plasma was challenged with 10 µL of the diluted bacterial solution. 50 µL of the plasma-bacteria mixture was immediately spread onto an agar plate as a 0 min sample. The remaining plasma-bacteria mixture was incubated at 25°C for 60 min. After 60 min, a further 50 µL sample was spread onto an agar plate. To quantify bacterial growth after 60 min in the absence of toad plasma, we also prepared nine control samples, in which 10 µL of dilute solution of each bacteria type was added to 140 µL of sterile CO_2_-independent media (in place of toad plasma), and plated out at 0 and 60 min. All *E. coli* plates were incubated at 37°C for 24 h and the number of colonies on each plate was counted by hand. *O. anthropi* plates were incubated at 30°C for 72 h, and the number of colonies counted. For each toad’s plasma sample and each bacteria type, we calculated a BKA index value according to Allen et al. [46].
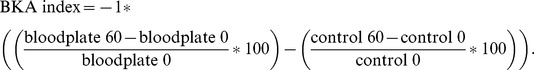



#### Phagocytosis chemiluminescence assay

We added 240 µL of a 1∶20 dilution of whole blood in sterile amphibian Ringers to duplicate wells of a 96-well microassay plate along with 30 µL of luminol solution and 10 µL of zymosan solution. Control wells contained 240 µL of the blood dilution and 30 µL luminol but with 10 µL amphibian Ringers instead of zymosan. Immediately upon addition of zymosan to the wells, the plate was placed in a luminometer (FluoroStar Optima; BMG Labtech, Ortenberg, Germany) and luminescence was read at 5-min intervals over 120 min.

#### Corticosterone stress response assay

Corticosterone concentrations in the first and second blood samples were measured using commercial enzyme immunoassay kits (Octeia Corticosterone HS, IDS Ltd, Boldon, UK) that have been validated for use on cane toads [47]. We refer to the corticosterone concentration measured from the first blood sample (collected within 3 min of the toads capture in the field) as the baseline level, and the concentration in the second sample (collected after the toad was held in the lab for 24 h) as the post-stress level. We used the difference in corticosterone levels between baseline and post-stress samples as an indicator of the toad’s stress response to being removed from the wild and held in captivity, under standard conditions. We were unable to collect post-stress blood samples from three toads within three minutes of removing them from holding bags, thus sample sizes for post-stress corticosterone levels and changes in corticosterone is 21, rather than 24.

#### PHA skin-swelling assay

On the morning after their return to the lab, and after their second blood sample was collected, each toad began a PHA assay [48]. The webbing between the second and third toes of the right hind foot was injected with 0.05 mL of a 2 mg/mL solution of PHA (Sigma L7854) in sterile PBS using a 29 gauge needle. As a control, the webbing between the toes of the left hind foot was injected with 0.05 mL of sterile PBS. The thickness of each injected toe web was measured prior to injection, and 6, 12, 24, 48 and 72 h after injection using a dial thickness gauge (Peacock G1-A; Ozaki Manufacturing Ltd, Tokyo, Japan). The relative amount of swelling caused by PHA, over and above that induced by injecting PBS alone, was calculated as:
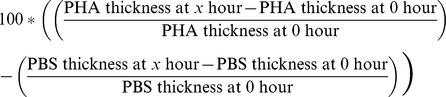



Because the PHA assay was initiated after the toads had been held in captivity for 24 h, and continued over a further three days of captivity, it could unavoidably be affected by stress-induced changes in corticosterone levels. The same standardized procedure was followed for all toads, so exposure to the stressor (captivity) was consistent.

### Statistical Analyses

We used two approaches to analyze the relationship between a toad’s movement distance and its immune function. Initially, we assessed each immune assay separately, using repeated-measures analysis for PHA and phagocytosis assays and multiple linear regressions for other dependent variables. We included two-way interactions among distance, sex and SUL in preliminary multiple regression models, but in all cases they were insignificant so they were removed from reduced models that contained only the three main effects. For the repeated-measures analyses our initial models included the two-way interactions among distance, sex and SUL as well as their interactions with time. Again, nonsignificant interaction orders were removed from the final reduced models.

Our second approach conglomerated immune measures using principal component (PC) analysis and used the PCs as dependent variables to represent overall immune function. We used the total distance that a toad moved over seven days radio-tracking as the explanatory variable of interest in all analyses, as well as sex and body size as potential covariates that may affect immune responses. All two-way interactions were included in preliminary models and nonsignificant interactions removed from final reduced models. We assessed all variables for deviations from assumptions of normality. Values for distance, as well as baseline and post-stress corticosterone level, were ln-transformed prior to analyses to normalize residuals. Multiple regressions and PC analysis were carried out using JMP (SAS Institute, Cary, NC), whereas repeated-measures analyses on PHA swelling and luminescence were carried out using Proc Mixed in SAS (SAS Institute, Cary, NC).

## Results

### Movements

At the end of seven days of telemetry we recovered 24 of the 30 radio-tracked toads to perform immune assays. Two of the toads were killed by predators during telemetry, two moved to inaccessible areas and were unable to be recaptured on schedule, the transmitter fell off one toad and the transmitter failed on another. Over the seven-day radio-tracking period, the average distance moved by the 24 toads was 995.4±203 m, but the total distance moved varied ten-fold among individuals, from 35 to 3506 m. Distance moved was independent of sex (*F*_1,21_ = 0.52, *P* = 0.47) and body size (*F*_1,21_ = 1.18, *P* = 0.29). Toads moved almost every day (on average, 6.3 out of 7 days). The toads’ movements were mainly dispersive: that is, they moved progressively further away from their original capture point. At the end of the seven-day tracking period, displacement from the initial capture point averaged 815.5 m (SE = 193.1, range 3.2–3003.2 m). Final displacement was highly correlated with total distance moved over the seven days (*r* = 0.99, *P*<0.0001). One radio-tracked toad had moved 5957 m and displaced 5395 m during the telemetry period, but we were unable to recapture her on the seventh day to perform immune assays.

### White Blood Cell Differentials

Lymphocytes and neutrophils were the most commonly observed white blood cells, comprising 50% and 36% of leukocytes, respectively. Basophils comprised 5.7% of white blood cells, eosinophils 5% and monocytes 3.2%. Neutrophil concentration was unaffected by sex and distance moved (both *F*_1,20_<1.16, both *P*>0.29), but did decrease significantly with body size (*F*_1,20_ = 4.66, *P* = 0.043). Lymphocyte concentration though, was unaffected by sex, body size or distance moved (all *F*_1,20_<0.66, all *P*>0.43). All other metrics derived from hemocytometry (neutrophil: lymphocyte ratio, concentrations of leucocytes, erythrocytes, basophils, eosinophils, and monocytes) were similarly unrelated to sex (all *F*_1,20_<1.80, all *P*>0.19), body size (all *F*_1,20_<1.83, all *P*>0.19) and distance moved (all *F*_1,20_<3.34, all *P*>0.083).

### Bacteria-killing Assay

Toad plasma significantly suppressed both types of bacteria. After 60 min incubation in the presence of toad plasma, the number of *E. coli* colonies decreased by an average of 59.5% and the number of *O. anthropi* colonies decreased by 11.3%. Colony counts of *E. coli* control samples (incubated for 60 min in the absence of toad plasma) only decreased by 1.4% for *E. coli*, whereas colony counts of *O. anthropi* control samples increased by 15.2%.

**Figure 3:**
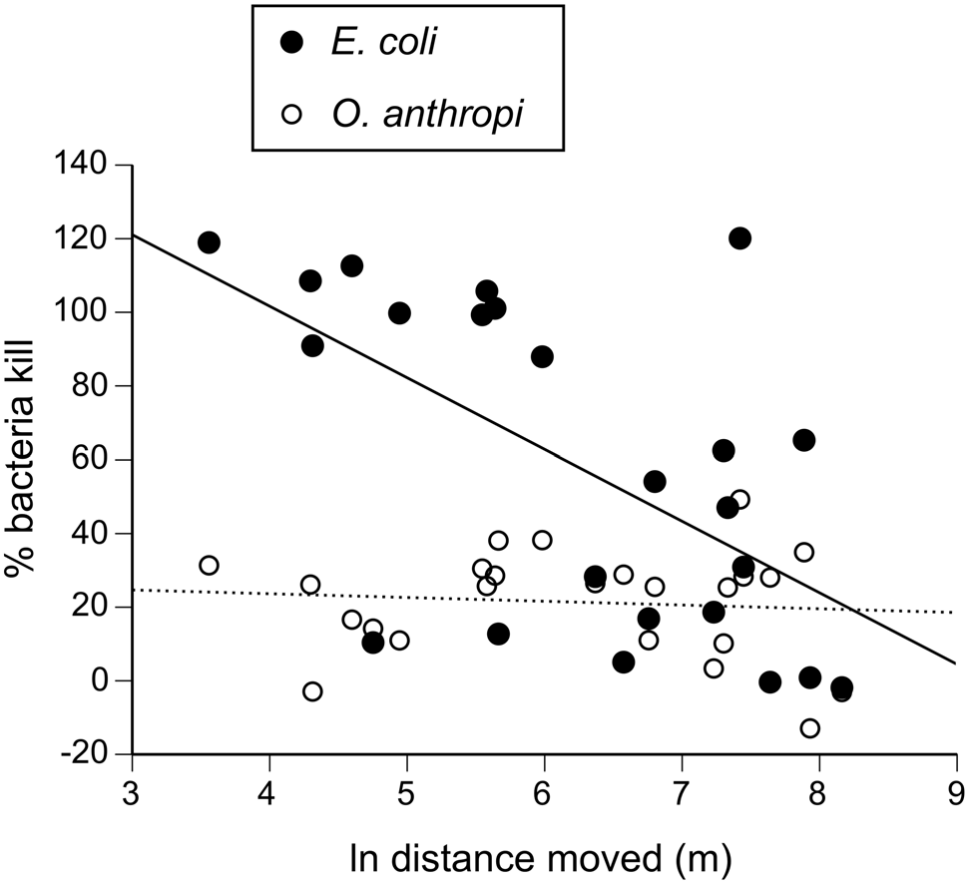
Bacteria-killing ability of toad plasma. After 60*E. coli* (closed, solid) plasma from toads that had moved further during the previous week was less bactericidal, than was plasma from more sedentary toads. The ability of toad plasma to kill *O. anthropi* (open, dashed) was unaffected by the distance the toad had moved.

When challenged with *E. coli,* the bacteria-killing ability of toad plasma was independent of sex and body size, but was negatively related to the distance the toad had travelled during the previous week (Table 1, Fig. 3). When challenged with *O. anthropi,* however, bacteria-killing was not related to sex, body size or distance moved (Table 1, Fig. 3).

**Table 1:** Effects of cane toad sex, body size (snout-urostyle length; SUL) and ln-transformed movement distance (during radio-tracking) on the ability of toad plasma to inhibit two bacteria types *in* *vivo*.

Bacteria	Variable	df	*F*	*P*
*E. coli*	Sex	1,20	0.61	0.44
	SUL	1,20	0.05	0.83
	ln distance	1,20	10.3	**0.004**
*O. anthropi*	Sex	1,20	0.10	0.33
	SUL	1,20	0.02	0.88
	ln distance	1,20	0.22	0.65

Bold font indicates *P*-values that are significant at α = 0.05.

### Phagocytosis Chemiluminescence Assay

**Figure 4:**
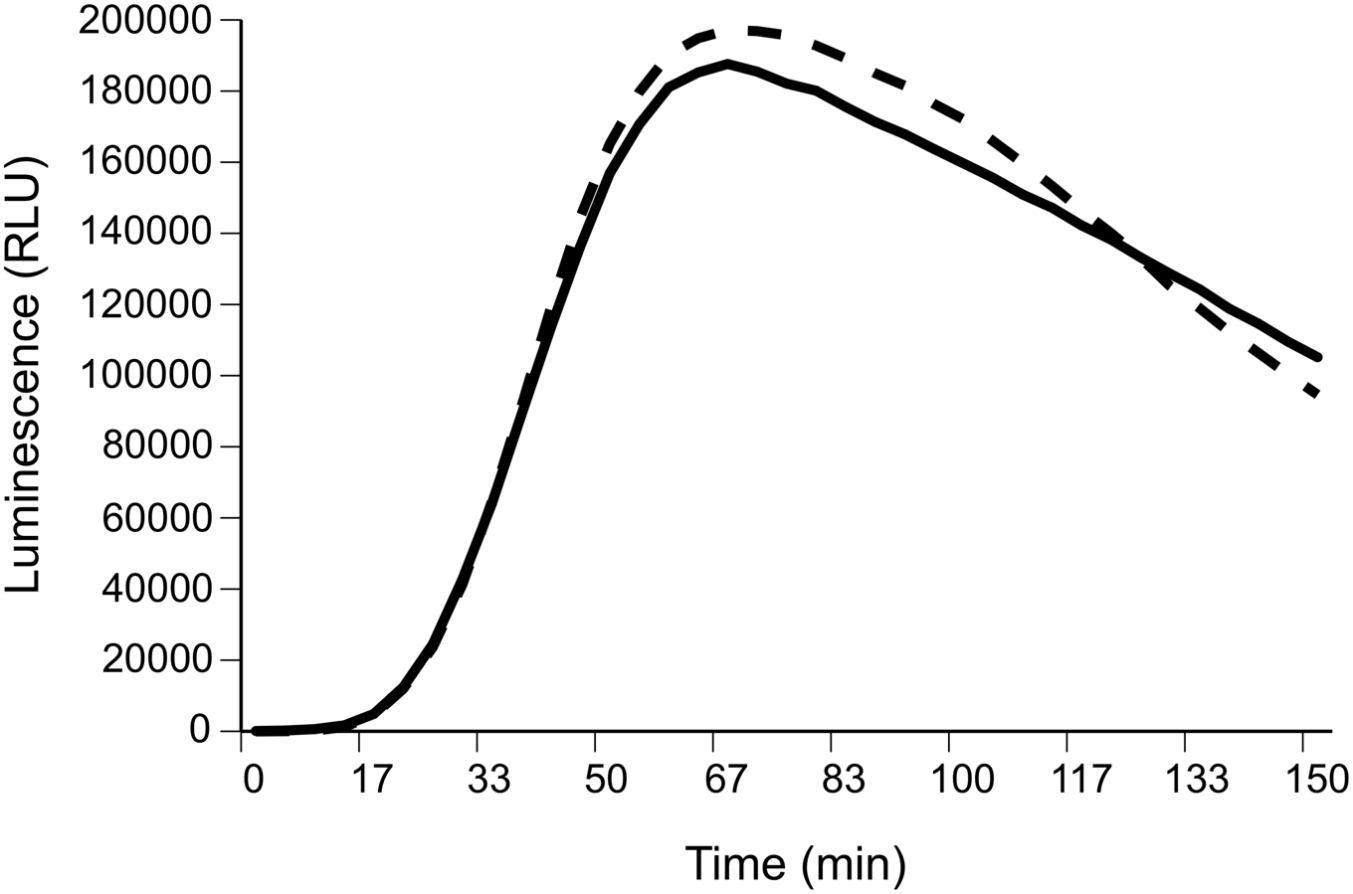
Phagocytic activity of toad whole blood. Luminescence (RUL = relative luminescence units) profiles of blood from toads that moved greater than the mean distance (995 m) (dashed line) and less than the mean distance (solid line). Distance groupings are for graphical purposes only; in our statistical analyses, we treated distance moved as a continuous variable.

Toads that had moved further during the previous seven days had less phagocytic whole blood. Repeated-measures analysis of luminescence readings over 120 min revealed a significant time effect and a significant interaction between time and ln distance moved (Table 2, Fig. 4). Sex, body size and their respective interactions with time were nonsignificant (Table 2, Fig. 4).

**Table 2:** Repeated-measures analysis of the effects of toad sex, body size (snout-urostyle length; SUL) and ln-transformed distance moved on phagocytic ability of whole blood, measured by luminescence.

Effect	df	*F*	*P*
Sex	1,20	0.91	0.3521
SUL	1,20	0.23	0.6377
ln distance	1,20	4.66	**0.0431**
Time	29,580	1.75	**0.0098**
Sex*time	29,580	0.33	0.9997
SUL*time	29,580	0.98	0.4987
ln distance*time	29,580	1.54	**0.0370**

Bold font indicates *P*-values that are significant at α = 0.05.

*indicates a statistical interaction between variables.

### PHA Skin-swelling Assay

**Figure 5:**
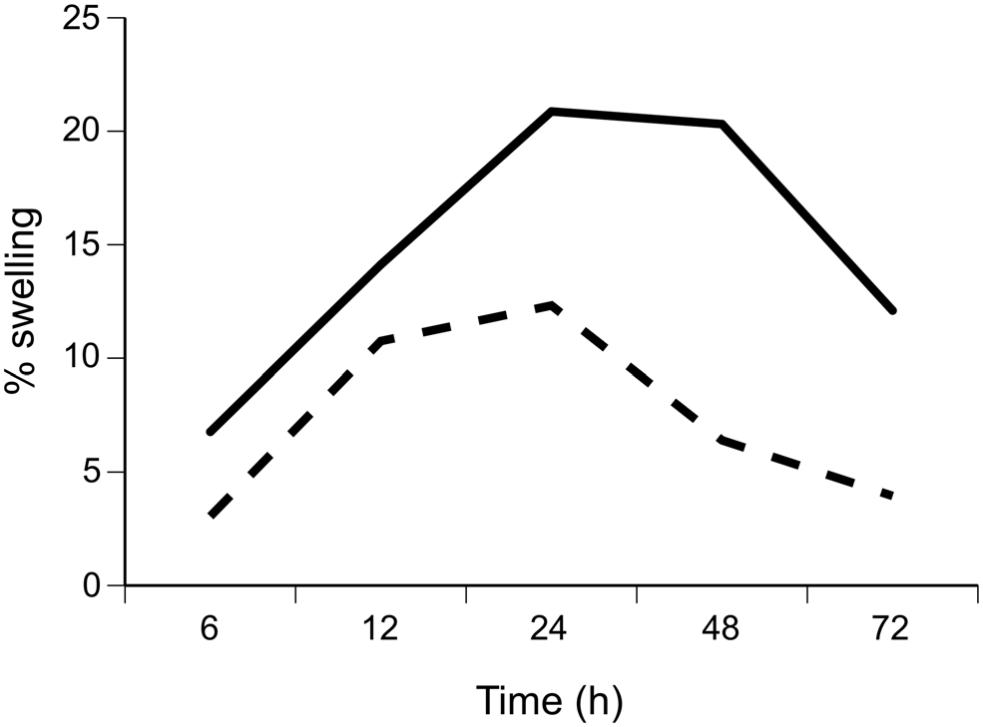
Skin swelling induced by phytohemagglutinin (PHA). Comparison between toads that had moved greater than the mean distance (995 m) (dashed line) and less than the mean distance (solid line) over the previous week. “% swelling” indicates the relative difference in web thickness between a toe web injected with PHA and a control web injected with saline. Distance groupings are for graphical purposes only; in our statistical analyses, we treated distance moved as a continuous variable.

Toads that moved further exhibited greater swelling in response to PHA injection. Repeated-measures analysis of the relative amount of swelling induced by PHA indicated a significant main effect of ln distance (Table 3, Fig. 5). There were no significant effects of toad sex, body size or time and no significant interactions (Table 3, Fig. 5).

**Table 3:** Repeated-measures analysis of the effects of toad sex, body size (snout-urostyle length; SUL) and ln-transformed dispersal distance on the relative amount of toe web swelling induced by PHA injection.

Effect	df	*F*	*P*
Sex	1,19	0.58	0.4575
SUL	1,19	1.86	0.1888
ln distance	1,19	5.89	**0.0254**
Time	4,19	1.08	0.3931
Sex*time	4,19	0.86	0.5039
SUL*time	4,19	0.98	0.4442
ln distance *time	4,19	1.44	0.2597

Bold font indicates *P*-values that are significant at α = 0.05.

*indicates a statistical interaction between variables.

### Corticosterone Stress Response Assay

Being held captive in a cloth bag for 24 h caused corticosterone levels of toads to increase from an average of 6.4 ng/mL to 18.2 ng/mL. Baseline corticosterone, measured from the blood sample collected immediately upon capturing the toad, was unrelated to sex or body size (*F*_1,20_<1.0, *P*>0.33). Although baseline corticosterone level tended to be higher amongst toads that had moved further, the effect was not significant (slope = 0.27, *F*_1,20_ = 2.67, *P* = 0.12). Post-stress corticosterone level, measured after 24 h in captivity, also was unrelated to sex, body size (*F*_1,17_<2.45, *P*>0.14) or distance moved (*F*_1,17_ = 0.35, *P* = 0.56). The proportional increase in corticosterone between baseline and post-stress samples was also independent of sex, body size and distance moved (*F*_1,17_<1.2, *P*>0.29). Baseline corticosterone levels were not correlated with any of the other immune measures, indicating that any variation within the immune measures was not regulated by corticosterone level (Table 4).

**Table 4:** Matrix of correlations among 14 measures of immune function in cane toads.

	BKA index of*E. coli*58.1±9.0	BKA index of*O. anthropi*21.4±3.0	Meanluminescence(RLU)117170±13047	Maximumluminescence(RLU)212508±25579	Time tomaximumluminescence (min)76.1±4.3	PHA %swelling at 6 h4.3±2.7	PHA %swellingat 12 h11.9±3.7	PHA %swellingat 24 h15.3±5.3	PHA %swellingat 48 h11.1±3.8	PHA %swellingat 72 h6.6±3.1	Neutrophils/mL4550±670	Lymphocytes/mL7218±1234	Baselinecorticosteroneng/mL6.4±2.2	Post-stresscorticosterone ng/mL18.2±3.6
BKA index of*E. coli*		0.36	**0.71**	**0.69**	**−0.49**	**−**0.14	**−**0.27	**−**0.19	**−**0.20	**−**0.20	0.06	0.03	**−**0.28	**−0.50**
BKA index of*O. anthropi*	0.083		0.32	0.30	**−0.41**	0.10	**−**0.08	0.08	0.04	**−**0.09	0.20	0.18	**−**0.31	**−**0.23
Meanluminescence	**0.000**	0.128		**0.99**	**−0.73**	**−**0.31	**−0.54**	**−**0.30	**−**0.37	**−**0.41	**0.44**	0.09	**−**0.21	**−**0.35
Maximumluminescence	**0.000**	0.156	**0.000**		**−0.73**	**−**0.34	**−0.52**	**−**0.32	**−**0.39	**−0.43**	**0.51**	0.17	**−**0.31	**−**0.37
Time tomaximumluminescence	**0.016**	**0.049**	**0.000**	**0.000**		0.14	0.30	0.10	0.16	0.32	**−**0.10	**−**0.05	0.15	0.38
PHA swellingat 6 h	0.532	0.656	0.148	0.107	0.509		**0.60**	**0.58**	**0.57**	**0.55**	**−**0.10	**−**0.01	0.38	**−**0.24
PHA swellingat 12 h	0.213	0.709	**0.009**	**0.011**	0.157	**0.003**		**0.80**	**0.78**	**0.79**	**−**0.32	0.14	0.17	**−**0.11
PHA swellingat 24 h	0.394	0.711	0.158	0.143	0.648	**0.003**	**0.000**		**0.62**	**0.53**	**−**0.05	0.35	0.24	**−**0.25
PHA swellingat 48 h	0.353	0.867	0.078	0.066	0.474	**0.004**	**0.000**	**0.002**		**0.78**	**−0.45**	0.06	0.16	**−**0.04
PHA swellingat 72 h	0.367	0.699	0.053	**0.043**	0.134	**0.006**	**0.000**	**0.010**	**0.000**		**−**0.29	0.03	0.24	0.20
Neutrophils/mL	0.770	0.337	**0.030**	**0.011**	0.655	0.644	0.133	0.813	**0.033**	0.178		**0.48**	**−**0.33	**−**0.16
Lymphocytes/mL	0.893	0.396	0.681	0.424	0.828	0.948	0.525	0.100	0.797	0.878	**0.017**		**−0.47**	**−**0.19
Baselinecorticosterone	0.189	0.137	0.320	0.138	0.474	0.072	0.431	0.265	0.461	0.264	0.117	**0.020**		0.26
Post-stresscorticosterone	**0.020**	0.316	0.125	0.095	0.087	0.306	0.650	0.286	0.880	0.408	0.491	0.398	0.263	

Values above the diagonal are Pearson coefficients and values below the diagonal are *P*-values. Bold font indicates coefficients and *P*-values that are significant at α = 0.05. Mean values and standard errors for each measure appear in the column headings.

### Multivariate Immune Response

We detected many significant correlations (both positive and negative) among the 14 different immune measures (Table 4). Some of these correlations are not surprising, as they occur between similar assays. For example, plasma samples that were proficient at suppressing one type of bacteria tended to suppress the other type as well. Similarly, mean and maximum luminescence values were highly correlated with one another, and were also highly correlated with the concentration of neutrophils in the blood, verifying the role that these cells play in phagocytosis. But we also found significant correlations between measures from different assays. Bacteria killing ability was correlated with measures of phagocytic ability, which in turn were negatively correlated with measures of skin swelling (Table 4). These correlations suggest that a multivariate approach (such as PC analysis) is warranted.

**Figure 6:**
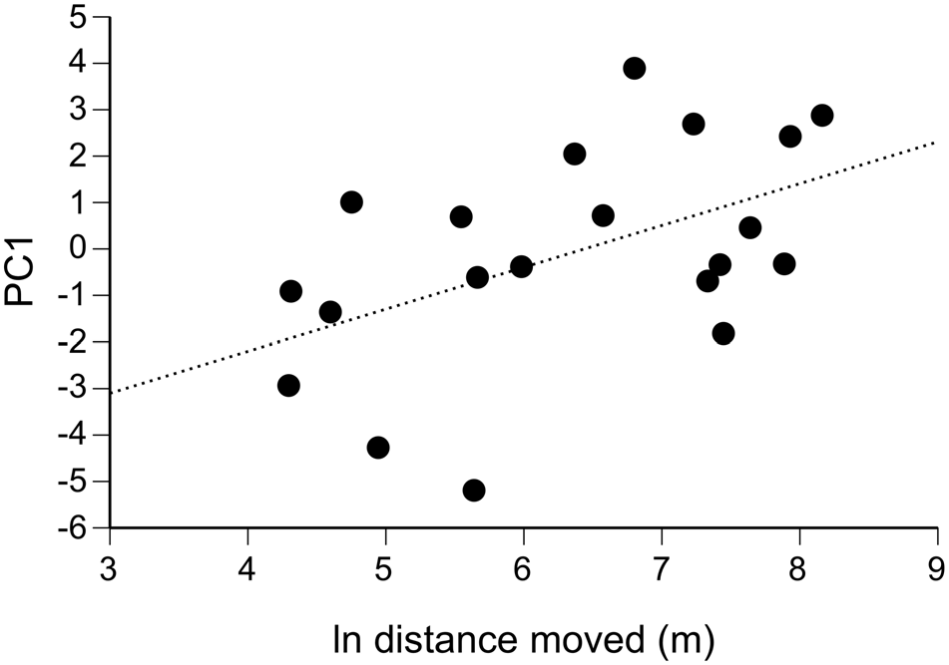
Relationship between a conglomerate immune measure and distance moved. Immune PC1 values of individual cane toads regressed on the total distances moved by those animals during seven days of prior radio-tracking. Positive values of PC1 indicate toads with poor bacteria-killing and phagocytosis scores, low neutrophil levels, and a large skin-swelling response to phytohemagglutinin.

The first three PCs from the analysis explained 70% of the variation among our 14 measures of immune function (Table 5). PC1 showed heavy loadings on all variables except for the BKA index for *O. anthropi*, and levels of corticosterone and lymphocytes. Positive values of PC1 described toads with strong PHA responses, poor bacteria-killing and phagocytic abilities and low neutrophil concentrations. Using the three PC’s as independent variables in multiple regression revealed that PC1 was positively related to distance moved (Table 6, Fig. 6) but not to a toad’s sex or body size (Table 6). PC2 and PC3 were not significantly related to any of the explanatory variables (Table 6).

**Table 5:** Principal component loadings formed by 14 measures of immune function in cane toads.

Effect	PC1 (37.7%)	PC2 (20.0%)	PC3 (11.9%)
BKA index *E. coli*	**−0.6**	0.4	**−**0.4
BKA index *O. anthropi*	**−**0.3	**0.5**	0.0
Mean luminescence	**−0.8**	0.3	**−**0.2
Maximum luminescence	**−0.9**	0.3	**−**0.1
Time to maximum luminescence	**0.6**	**−**0.4	0.4
PHA swelling at 6 h	**0.6**	**0.5**	**−**0.2
PHA swelling at 12 h	**0.8**	**0.5**	0.0
PHA swelling at 24 h	**0.6**	**0.6**	0.1
PHA swelling at 48 h	**0.7**	**0.5**	**−**0.2
PHA swelling at 72 h	**0.7**	0.3	**−**0.1
Neutrophils/mL	**−0.5**	0.2	**0.6**
Lymphocytes/mL	**−**0.1	**0.5**	**0.8**
Baseline corticosterone	0.4	**−**0.2	**−0.5**
Post-stress corticosterone	0.3	**−0.7**	0.1

Bold font is used to indicate relatively heavy loadings (>0.5). Values in parentheses under headings indicate the amount of total variation explained by that PC.

**Table 6:** Results of multiple regression models of the effects of toad sex, body size (snout-urostyle length; SUL) and ln-transformed distance moved on the first three immune PCs.

	Effect	df	*F*	*P*
PC1	Sex	1,19	0.48	0.50
	SUL	1,19	0.84	0.37
	ln distance	1,19	6.00	**0.026**
PC2	Sex	1,19	0.10	0.76
	SUL	1,19	0.66	0.43
	ln distance	1,19	0.17	0.68
PC3	Sex	1,19	0.11	0.74
	SUL	1,19	3.37	0.09
	ln distance	1,19	0.08	0.78

Bold font indicates *P*-values that are significant at α = 0.05.

## Discussion

Our study site is 1700 km from the point where toads were introduced into Australia. They dispersed this distance in 70 years, passing through a wide variety of landscapes, climates, and native fauna and pathogens. Surveys of parasites and diseases in Australian cane toads provide some indication of the pathogen pressure faced by invading toads. At least one of the 100 toads brought to Australia from Hawaii in 1935 harbored a nematode lungworm native to South America [49] and this parasite is now common in Australian toad populations [50], though it lags behind the frontline of the toad invasion, where host density is too low for transmission [35]. Aside from harboring and outrunning a lungworm with which they have a long association, toads in Australia are also exposed to and can become infected with a suite of native pathogens [51]–[53], including pentastomids [37], myxosporea [54], and native nematode lungworms (GPB unpublished data).

Although toads have inhabited our study site for eight years, telemetry and mark-recapture data suggest that the population consists entirely of dispersive rather than resident individuals. Rates of dispersal have decreased since the first wave of invasion [55], but the current study illustrates that the toads still show remarkable rates of movement. The dispersal abilities of amphibians are often under-estimated, and displacements of several kilometers are likely possible among many species, over months or years [2], [56]. Displacing several kilometers in a matter of days however (as regularly observed among cane toads at our study site) may be the highest movement rate recorded for any amphibian [40], [57]. Considering that their body length is only 100 mm, it is remarkable that some of the toads in this study moved up to 1251 m over land (as opposed to through water), sometimes through dense ground cover, in one night. A sprinting toad covers about 2.5 body lengths per hop, so a 100 mm toad needs to hop >5000 times to cover 1251 m. Importantly, other toads remained much more sedentary, moving as little as 35 m in seven days. Thus, our study incorporated toads whose movements varied dramatically (10-fold), allowing us to assess immune responses over a wide range of activity levels.

The immune responses of toads that moved longer distances over the seven-day telemetry period differed from those of their more sedentary conspecifics. Both the analyses of individual immune measures, and of our composite (PC) scores, showed that toads that moved further had poorer bacteria-killing and phagocytic abilities, but exhibited greater skin-swelling in response to PHA injection. Greater displacement distances were not significantly associated with higher corticosterone levels, either baseline or in response to captivity stress.

Are the changes in immune function of the most mobile toads an adaptive response to tissue damage arising from strenuous exercise, or the result of an energetic trade-off? The former scenario would predict (1) a positive correlation between distance moved and baseline corticosterone level, and (2) a negative relationship between measures of immune function and baseline corticosterone level. Neither of these relationships were evident in our data. If the down-regulation in bactericidal and phagocytic ability is an adaptation to avoid autoimmune reactions, it must be regulated by something other than corticosterone. Another argument against a general down-regulation (either adaptive or nonadaptive [31]) of immune function following physical activity is the observation that PHA-response was enhanced, not subdued, amongst the toads that moved furthest.

The finding that long-distance movement induced down-regulation of some immune functions and up-regulation of another is more consistent with a trade-off scenario, wherein investment in different components of the immune system is altered in response to the energetic or nutritional requirements of sustained activity. Because we only assessed a small component of the toads’ immune arsenal, we cannot make definitive statements about where the observed shifts in investment have placed the toads along a ‘protection vs. cost’ continuum. However, the decreased bacteria-killing and phagocytic ability of the most mobile toads bears directly on a pathological condition that is common in cane toads at the invasion front. Toads from invasion-front populations exhibit high rates of septic (as opposed to autoimmune) spinal arthritis [21]. This spinal arthropathy results from high activity levels, which cause erosion of intervertebral surfaces. These surfaces become seeded with the bacterium *O. anthropi* and the subsequent inflammation produces bony proliferation and fusion of the associated discs [53] (hence our choice to include *O. anthropi* as a bacterial challenge in the present study). Thus, the high rates of spinal arthropathy in invasion-front toads may be facilitated by the general decrease in bactericidal and phagocytic abilities associated with high rates of movement, as well as the resultant increased wear and tear on articular spinal surfaces. Toad plasma is relatively inefficient at killing *O. anthropi*, so other lines of defense such as phagocytes or antimicrobial skin secretions may be more important for controlling its establishment.

Significant negative correlations between some components of immune function (e.g. PHA responses vs. phagocytic responses) suggest that trade-offs occur within the immune repertoire of free-ranging toads. The axis that best describes the intrapopulational variation in immune response is one that combines strong cell-mediated function with weak bactericidal/phagocytic function at one end and the converse at the other. We cannot evaluate whether one of these contrasting immune architectures represents a more economical strategy than the other, nor that one confers greater immunocompetence than the other. The major cost associated with a given immune response is not necessarily its establishment or maintenance, but rather the costs incurred if its activation induces a systemic inflammation response [34]. A systemic inflammatory response can be triggered by either innate immune responses (such as bacterial lysis or phagocytosis) or by cell-mediated immune responses (as assayed by PHA-induced swelling) [18], [58] and is accompanied by dramatic metabolic and behavioral changes (e.g. increased oxygen consumption, lethargy, anorexia) [59], [60]. In our toads, indicators of one of these general triggering mechanisms (innate) were down-regulated, while an indicator of the other (cell-mediated) was enhanced. Therefore, there is no clear evidence that the most active toads reduce their immune investment to avoid costly systemic inflammation reactions. The prediction that toads that move long distance are less likely to mount a systemic inflammation reaction in response to an immune challenge is readily testable.

In humans, a causal link between strenuous activity and immune function is well established. Rigorous exercise results in changes in leukocyte concentration, distribution and function, and resultant tissue damage can initiate inflammatory cytokine cascade [5], [7], [8]. Although we found a strong association between a toad’s behavior (the distance it moved during radio-tracking) and its physiology (immune function), our data are correlational and hence, we cannot be sure of causality. Thus, the differences in immune response among our toads could have been associated with the causes rather than consequences of the animal’s movement patterns. In birds, migratory restlessness (in the absence of the actual physical and energetic demands of migration) affects immune function [36] more than does strenuous exercise *per se* [15]. Conceivably, toads preparing for long bouts of dispersal might undergo physiological and behavioral changes; and/or individuals with specific types of immune function might somehow be more likely to move in specific ways. Alternatively, the differences in immune response seen among our toads could have arisen subsequent to (i.e. as a result of) the physical exertions of movement.

Further study is needed to distinguish between the above alternatives, because they have important implications. For example, changes in immune function that occur prior to dispersal could be evolved responses to the invasion process, and represent a correlated suite of dispersive genotypes (morphological, behavioral, physiological and immunological) that have arisen through genetic drift, spatial sorting and/or natural selection [20]. Toads that are behaviorally predisposed to move long distances also may have immune systems that are biased towards strong cell-mediated function and poor bactericidal/phagocytic function. Such an immune configuration may allow enhanced dispersal rates, but at the cost of an increased incidence of spinal arthropathy. On the other hand, if the changes in the immune responses that we observed are a result (rather than a cause) of increased muscle activity, and represent perturbation rather than adaptation, they might be a target for contemporary selection. For example, the physical act of dispersing may cause suppression of some immune functions in ways that save energy or avoid autoimmune reactions. But if this suppression results in increased rates of mortality or morbidity, then balancing selection may act against the immune down-regulation.

Regardless of how the changes in immune response of the most active, dispersing toads arise (whether induced or canalized), the implications are important if they can be extended to invasive fauna in general. The individuals comprising the vanguard of an invasion or range-expansion may have an altered immune system compared to individuals in the native range or range core. Thus, they may be more susceptible to pathogens, or susceptible to more pathogens, than are sedentary conspecifics. Also, individuals at the edge of an expanding range may ‘outrun’ pathogens that are common in the range core [35]. Escape from such a pathogen, through range expansion, may be an important factor in any subsequent trade-offs among immune components. Translocating a native pathogen from the core range into the path of the invasion front might offer a means of slowing the spread if it has a more pronounced pathological effect on any individuals at the range edge that have traded away their ability to resist it [32].

Alternatively, a change in immune response during range expansion or invasion may favor increased levels of tolerance to pathogens as well as decreased resistance [3]. Release from enemies can result in increased tolerance of herbivory in invasive plants [61], [62]. Extending this example to animals [63], the changes in lifestyle and energy budgets attendant to range expansion could result in increased pathogen tolerance among invasive species [3]. Individuals able to forego the expense (e.g. energy, time, anorexia) of a systemic inflammation response to pathogen attack would have more time and energy for dispersal [58]. In cane toads, individuals originating from closer to the invasion front showed a smaller increase in metabolic rate in response to a standardized immune challenge (lipopolysaccharide) [59].

Although alteration of chemical defenses during the process of invasion has long been recognized in plants, the analogous situation of altered immune defenses in animals has been recognized only recently [18], [19]. Much of the focus of this new field has involved the circumstance of enemy-release, a common consequence of translocation [33], [34]. However, animal invasions may also entail the evolution of increased dispersal ability, and dramatically increased rates of movement [64]–[66]. The results of our correlational study suggest that interactions between the immune system and elevated rates of movement could have important influences on the ecoimmunology of invasion and range expansion.
